# Editorial: The inflammation markers in schizophrenia and bipolar disorder: Do we have promising results?

**DOI:** 10.3389/fpsyt.2022.1128355

**Published:** 2023-01-05

**Authors:** Ayla Arslan, Orkun Aydin, Fikret P. Çökmüş

**Affiliations:** ^1^Department of Molecular Biology and Genetics, Faculty of Engineering and Natural Sciences, Üsküdar University, Istanbul, Turkey; ^2^Genetics and Bioengineering Program, Faculty of Engineering and Natural Sciences, International University of Sarajevo, Sarajevo, Bosnia and Herzegovina; ^3^Department of Psychology, Faculty of Arts and Social Sciences, International University of Sarajevo, Sarajevo, Bosnia and Herzegovina; ^4^Department of Psychiatry, Faculty of Medicine, Izmir Tinaztepe University, Izmir, Turkey

**Keywords:** schizophrenia, bipolar disorder, inflammation, biomarker, diagnostic tests

Schizophrenia (SCZ) and bipolar disorder (BD) are multifactorial diseases with unknown precise etiology. The association of these diseases with multiple heterogeneous risk factors such as genetic variation, environmental, and epigenetic risk factors (1, 2) have led to the question to ask if a specific combination of these risk factors manifest the disease state and indices. As immune-inflammation response is an essential component of multifactorial disease pathogenesis (3), alterations in immune-inflammation response across the course of these two major psychiatric disorders have become critical. This is further supported by the accumulating evidence for the differential effects of anti-inflammatory medications on the treatment of mental disorders (4). Thus, the present Research Topic aims to examine the aspects of the immune-inflammation response conditions altered in the patients of SCZ and BD. This includes the association of number and activity of molecular and cellular inflammatory parameters with various disease states such as severity of disease symptoms often accompanied by biochemical, metabolic, or structural measures. In addition, as inflammation may also trigger extreme oxidative stress, the relevant parameters and their link to specific disease states is also included.

In attempt to determine the relation between inflammatory biomarkers (CRP, Eotaxin, Fractalkine, IP10, IL6, IL10, ICAM1, IFNγ, MCP1, MIP1β, SAA, TNFα, VEGF, and VCAM1) and cognitive and negative symptoms in SCZ, Klaus et al. have shown that the brain predicted age difference, i.e., the difference between brain age and chronological age, was higher in SCZ patients (*n* = 26) compared to the control group (*n* = 28) and these results were associated with higher peripheral levels of pro-inflammatory cytokine TNFα. Thus, patients with higher peripheral levels of TNFα had higher levels of advanced brain age. On the other hand, there was not any cognitive or negative symptom relationship with advanced brain aging and the inflammatory markers tested. Results of Yan et al. suggest that cytokines CRP, IL-8, IL-6, IL-13, and IL-16 as well as levels of white blood cell count, neutrophil count, natrium were higher in 79 first-episode drug-naïve patients with SCZ compared to 36 healthy controls. Arabska et al. examined the cytokine [CX3CL1 (Fractalkine), CXCL8, and IL-10] serum level and their patterns of cellular production in peripheral blood mononuclear cells obtained from the patients of SCZ (*n* = 60) and healthy controls (*n* = 32). Results show the differential correlation of these parameters with SCZ. Also, the differential effect of mood stabilizers (lamotrigine and valproate) on the serum concentration of CXCL8, fractalkine, and IL-10 was observed. Li et al. reported that the reduced activity of the Toll-like receptor 4 (TLR4) signaling pathway, the first line of defense against infections, was associated with white matter integrity and cognition deficits in a subject of 44 patients with stable chronic SCZ compared to 59 healthy controls. Altogether, these studies highlight the translational potential of inflammatory parameters regarding the disease etiology, diagnosis, prognosis, and drug response. Nevertheless, the small sample size is a major caveat to these studies.

On the other hand, one study appears to circumvent the limitation of the small sample size mentioned above. In an extensive analysis of the data collected from 13,329 patients with SCZ, 4,061 patients with BD manic episodes (BD-M), and 1,944 patients with BD depressive episodes (BD-D), and 5,810 healthy subjects, Wei et al. have reported the largest study to date that examined the peripheral inflammation parameters (systemic immune-inflammation index, neutrophil/high-density lipoprotein ratio, lymphocyte/high-density lipoprotein ratio, and monocyte/high-density lipoprotein ratio) in SCZ and BD. Results show the effect of inflammation on the pathophysiology of SCZ, BD-M, and BD-D. Interestingly, a relationship between the inflammation and lipid metabolism with differential association patterns in SCZ, BD-D, and BD-M was also described. Thus, the data suggest potential biomarkers for differential diagnosis.

Inflammation may also trigger an extreme oxidative stress, the overproduction of reactive oxygen species in the lack of sufficient antioxidants (5, 6). Thus, Yang et al. investigated whether there was an association between plasma oxidative stress markers [malondialdehyde (MDA), manganese superoxide dismutase (MnSOD), catalase (CAT), and glutathione peroxidase (GSH-Px)] and cognitive impairment among 96 patients (male) of chronic SCZ, with characteristics of long-term hospitalization. The plasma CAT and GSH-Px activities were associated with general pathological symptoms and visuospatial/constructional skills of SCZ patients. While these results partially confirmed the previous findings, the study highlights the need for more studies in this field to better understand the immune-inflammation response and associated mechanisms in SCZ and BD.

In conclusion, the current Research Topic provides an insight into our limited understanding of immune- inflammation response in mental disorders and highlights the correlation between inflammation and the various aspects of SCZ and BD. However, the findings generate new questions, such as those related to confounding factors and reverse causality. Thus, future studies require special attention on ruling out confounding factors and specific analytical methods to identify the cause and effect relationships in the context of inflammation and the pathogenesis of SCZ and BD.

## Author contributions

All authors listed have made a substantial, direct, and intellectual contribution to the work and approved it for publication.
